# Supplementary material for: MetaRibo-Seq measures translation in microbiomes
Source: Nat Commun. 2020 Jun 29;11:3268. doi: 10.1038/s41467-020-17081-z (PMC7324362; doi:10.1038/s41467-020-17081-z)
Supplement: Supplementary file 10 — Supplementary Data 7 [file 41467_2020_17081_MOESM10_ESM.zip › File2/Confidence_High_Taxonomy/119251_out.krona.html]

Javascript must be enabled to view this page.

members
magnitude
magnitudeUnassigned
count
unassigned
taxon
rank

119251\_out

3

superkingdom
3
2759

4751
kingdom
3

1
subkingdom
451864

SRS076929\_contig\_number\_contig-100\_3912.124987
3

4890
1
phylum

147538
1
subphylum

class
1
147550

1
subclass
222543

order
1
5125

110618
family
1

1
genus
5506

569360
1
species group

101028

SRS098571\_contig\_number\_51925
1
species

1
phylum
5204

subphylum
1
452284

1
class
1538075

1
order
162474

1
family
742845

55193
genus
1


SRS051031\_contig\_number\_contig-100\_5150.153736
1
species
55194
